# Supplementary material for: A pan-cancer analysis of homeobox family: expression characteristics and latent significance in prognosis and immune microenvironment
Source: Front Oncol. 2025 Feb 6;15:1521652. doi: 10.3389/fonc.2025.1521652 (PMC11840236; doi:10.3389/fonc.2025.1521652)
Supplement: Supplementary Figure 1 — Mutations of each HOX gene in 32 cancers (TCGA, PanCancer Atlas) by cBioportal. [file DataSheet1.zip › Suppl.files/raw code.zip!/code/4.survival/survivalplot.zip!/survivalplot/HOXD3_OS.LGG.pdf]

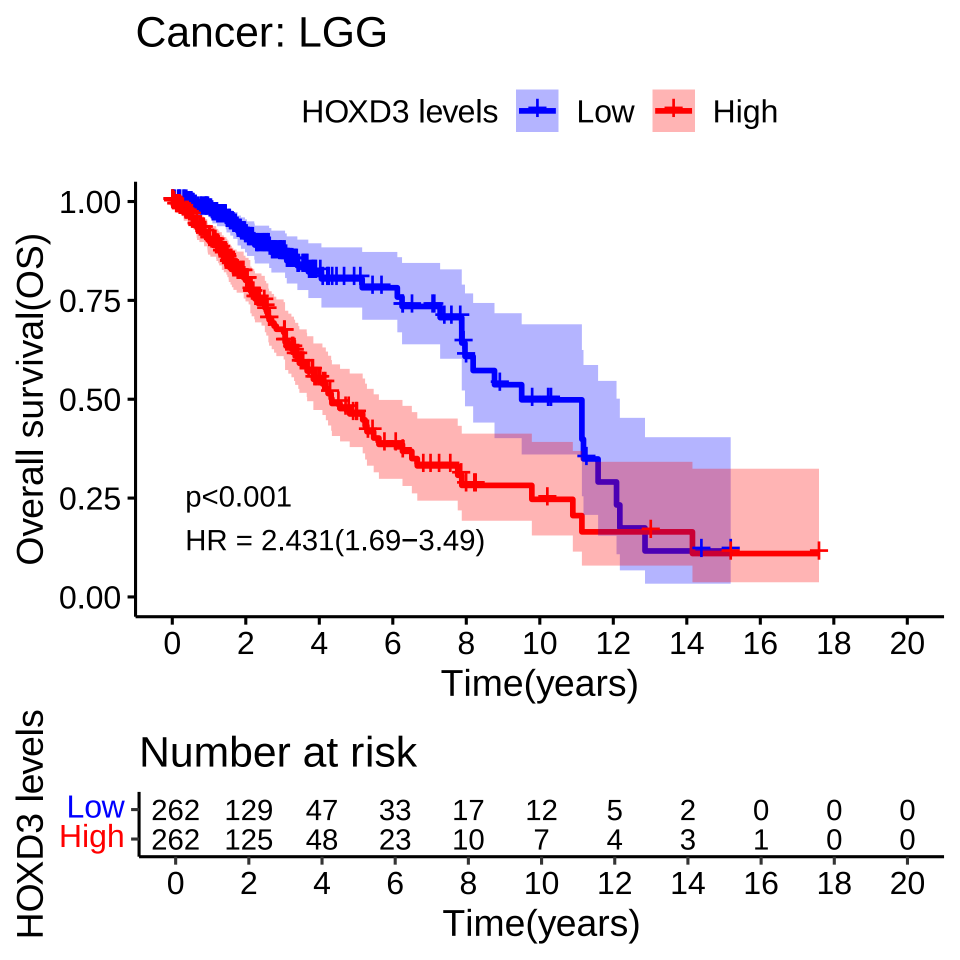
Cancer: LGG
HOXD3 levels
Low
High
1.00
0.75
0.50
0.25
0.00
Overall survival(OS)
p<0.001
HR = 2.431(1.69−3.49)
0 2 4 6 8 10 12 14 16 18 20
Time(years)
Number at risk
HOXD3 levels
Low 262 129 47 33 17 12 5 2 0 0 0
High 262 125 48 23 10 7 4 3 1 0 0
0 2 4 6 8 10 12 14 16 18 20
Time(years)
